# Long-term surveillance provides real-world evidences of safety and effectiveness in intravitreal aflibercept treatment for age-related macular degeneration

**DOI:** 10.1038/s41598-023-37584-1

**Published:** 2023-06-30

**Authors:** Yoko Ozawa, Kazuhiro Ohgami, Koji Sasaki, Kazufumi Hirano, Toshiyuki Sunaya

**Affiliations:** 1 Department of Clinical Regenerative Medicine Eye Center, Fujita Medical Innovation Center Tokyo, Fujita Health University School of Medicine, Tokyo, Japan; 2 Department of Ophthalmology, St. Luke’s International Hospital, Tokyo, Japan; 3 Department of Ophthalmology, St. Luke’s International University, Tokyo, Japan; 4 Department of Ophthalmology, Keio University School of Medicine, Tokyo, Japan; 5 Medical Affairs and Pharmacovigilance, Bayer Yakuhin, Ltd., Osaka, Japan; 6 Research and Development Japan, Bayer Yakuhin, Ltd., Osaka, Japan

**Keywords:** Retinal diseases, Medical research

## Abstract

This prospective, multicentre, postmarketing surveillance were conducted to report on the long-term safety and effectiveness of intravitreal aflibercept (IVT-AFL) treatment in clinical practice of Japanese patients with neovascular age-related macular degeneration (nAMD) who newly initiated IVT-AFL treatment. The primary outcomes were the incidence of adverse events (AEs) and of adverse drug reactions (ADRs) over 36 months. Number of injections, timing of ADR occurrence, and some effectiveness index were also summarised. A total of 3,872 patients received 7.2 ± 5.8 (mean ± standard deviation) injections, and AEs occurred in 5.73% of patients. ADRs were reported in 2.76% of patients, with ocular and nonocular ADRs in 2.07% and 0.72% of patients, respectively. Most vitreo-retinal events developed within 6 months of initial IVT-AFL treatment, and most instances of increased intraocular pressure and cerebral infarction developed after 6 months of follow-up. Mean best-corrected visual acuity and central retinal thickness were numerically better throughout the follow-up period compared with baseline. These results indicated acceptable tolerability and effectiveness of IVT-AFL treatment in patients with nAMD in clinical practice in Japan. Information regarding the risk and the timing of ADRs is valuable for safe and effective long-term treatment of patients with nAMD.

Trial registration number: NCT01756248.

## Introduction

Age-related macular degeneration (AMD) is one of the leading causes of vision impairment in adults aged 50 years or older worldwide^1^. The prevalence of AMD in the Japanese population aged 40 years or older is reported to be 1.2% and is increasing^2,3^. The advent of intravitreal anti-vascular endothelial growth factor (VEGF) therapy has greatly improved the visual prognosis in eyes with neovascular AMD (nAMD); however, this chronic and progressive disease is still an important cause of visual impairment in recently ageing societies^1,4^.

Aflibercept (Eylea, Regeneron, Tarrytown, NY, and Bayer HealthCare, Berlin, Germany) is an anti-VEGF agent that has high binding affinity to VEGF-A and can also bind to VEGF-B and placental growth factor^5^. Following confirmation of the clinical efficacy and safety of intravitreal aflibercept (IVT-AFL) treatment in patients with nAMD in two Phase III trials, VIEW 1 and 2^6,7^, IVT-AFL treatment has been extended to more diverse populations than in the VIEW trials, such as patients who have better best-corrected visual acuity (BCVA) at baseline^8,9^, and to longer treatment periods. Accordingly, the long-term safety and effectiveness of this treatment should be evaluated in a large cohort from routine clinical practice.

Based on the results from the VIEW trials^6,7^, the Japan risk management plan (RMP) identified some ocular events as ‘important risks’ and arterial thromboembolic events (ATEs) as ‘important potential risks’ that may occur during IVT-AFL treatment (supplemental Table 1)^10^. In addition, intraocular inflammation has been reported as an adverse drug reaction (ADR) of some intravitreal anti-VEGF agents^11–14^.

**Table 1:** Patient characteristics at baseline.

	Safety analysis set (n = 3,872)
Male sex, n (%)	2,650 (68.4)
Age, years	
Mean ± SD (range)	74.3 ± 8.9 (24–97)
< 40, n (%)	3 (0.1)
40–49, n (%)	24 (0.6)
50–59, n (%)	201 (5.2)
60–69, n (%)	808 (20.9)
70–79, n (%)	1,696 (43.8)
80–89, n (%)	1,060 (27.4)
≥ 90, n (%)	80 (2.1)
nAMD subtype*, n (%)	
tAMD	1,839 (47.5)
PCV	1,564 (40.4)
RAP	141 (3.6)
Types of CNV lesions on FA*, n (%)	
Occult CNV	1,228 (31.7)
Predominantly classic CNV	670 (17.3)
Minimally classic CNV	404 (10.4)
Other or unknown	1,576 (40.7)
Duration of disease from onset to first IVT-AFL, months	
Mean ± SD (range)	23.2 ± 32.6 (0–360)
< 2, n (%)	324 (8.4)
≥ 2, < 6, n (%)	290 (7.5)
≥ 6, < 12, n (%)	173 (4.5)
≥ 12, n (%)	747 (19.3)
Unknown	2,338 (60.4)
Best-corrected visual acuity, logMAR, n (%)	
< 0.301	1,614 (41.7)
≥ 0.301	2,238 (57.8)
Best-corrected visual acuity, logMAR, n (%)	
≤ 0.155	1,290 (33.3)
> 0.155	2,562 (66.2)

*Including overlap. *CNV* Choroidal neovascularization, *FA* Fluorescein angiography, *IVT-AFL* Intravitreal aflibercept, *nAMD* Neovascular age-related macular degeneration, *PCV* Polypoidal choroidal vasculopathy, *RAP* Retinal angiomatous proliferation, *SD* Standard deviation, *tAMD* Typical age-related macular degeneration.

In this study, we report on prospective observational surveillance that was performed to evaluate the safety profile and effectiveness of IVT-AFL treatment in patients with nAMD during 36 months of follow-up in Japan. Patient selection and treatment regimen were determined by the treating clinician. Thus, the results reflect those of routine clinical practice in Japan and may be informative for clinicians for the safe and effective management of patients over longer time periods.

## Results

### Patient population

A total of 4,428 patients who newly initiated IVT-AFL treatment (with or without prior treatments other than IVT-AFL) for nAMD were enrolled, of which 3,872 were included in the safety analysis set (SAF) and 3,684 in the effectiveness analysis set (EAS) (supplemental Figure 1).

**Figure 1:**
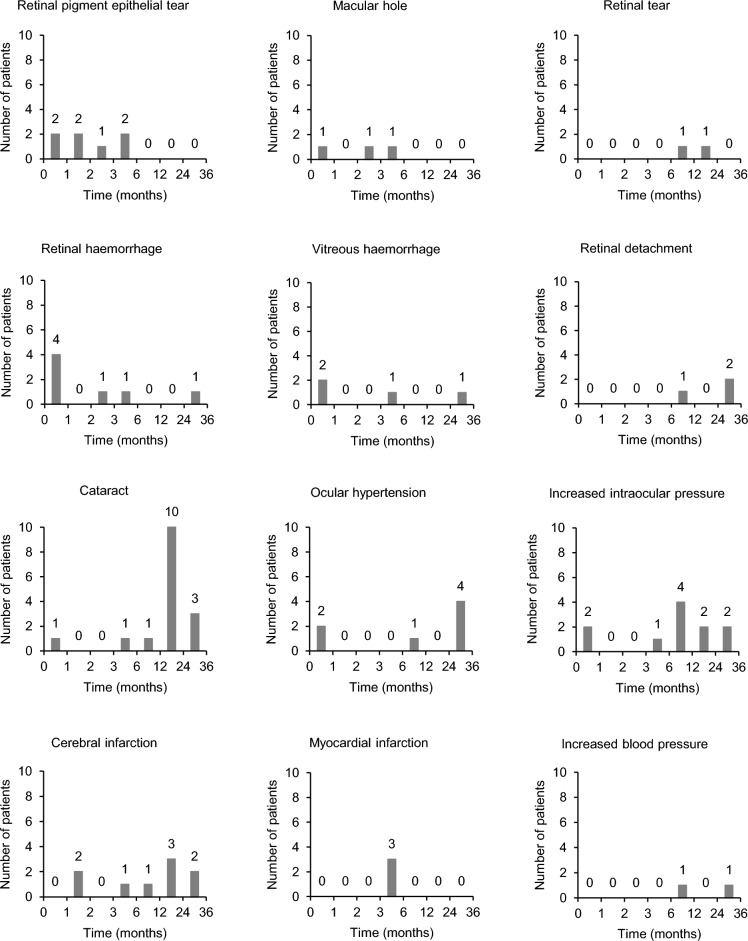
Timing of onset of ADRs. The number of patients who developed each ADR are shown for each period of + 30 days from the first date of IVT-AFL, + 31 to + 60 days, + 61 to + 90 days, + 91 to + 180 days, + 181 to + 360 days, + 361 to + 720 days, and + 721 to + 1,080 days. The period of any prior treatment was not considered. *ADR* adverse drug reaction, *IVT-AFL* intravitreal aflibercept.

Baseline characteristics of the 3,872 patients in the SAF are shown in Table 1. Age was 74.3 ± 8.9 (mean ± SD, range 24–97) years, with 2,836 patients (73.2%) aged 70 years or older, and 2,650 patients (68.4%) were male. Overall, 1,564 patients (40.4%) had polypoidal choroidal vasculopathy (PCV), and 41.7% of patients had baseline BCVA better than 0.301 logMAR (20/40 in Snellen’s chart, which was the upper boundary value for inclusion criteria in the VIEW trials) and 33.3% had baseline BCVA better than 0.155 logMAR (20/30 in Snellen’s chart, which is the visual acuity required to obtain a driver’s license in Japan). In the SAF, 1,309 patients (33.8%) had at least 1 ocular comorbidity at baseline, such as cataract (27.6%), glaucoma (4.0%), or conjunctivitis (2.6%), and 1,391 (35.9%) patients had at least 1 nonocular comorbidity, such as hypertension (24.3%), diabetes mellitus (8.5%), or dyslipidaemia (6.1%) (supplemental Table 2).

**Table 2:** Incidence of ocular and nonocular adverse events and adverse drug reactions.

Safety analysis set (n = 3,872)	Patients, n (%)			
	AE	SAE	ADR	SADR
Total events	222 (5.73)	94 (2.43)	107 (2.76)	44 (1.14)
Ocular event	159 (4.11)	45 (1.16)	80 (2.07)	23 (0.59)
Nonocular event	70 (1.81)	50 (1.29)	28 (0.72)	21 (0.54)

*AE* Adverse event, *ADR* Adverse drug reaction, *SADR* Serious adverse drug reaction, *SAE* Serious adverse event.

In the SAF, 1,222 patients (31.6%) had a history of at least 1 ocular medical condition, such as cataract (26.8%), and 890 patients (23.0%) had a history of at least 1 nonocular medical condition, such as hypertension (8.4%) (Supplemental Table 3). In addition, 2,519 patients (65.1%) had not been previously treated for nAMD (treatment-naïve patients) and 1,281 patients (33.1%) had been previously treated for nAMD with agents other than IVT-AFL (treatment-switched patients). Of the 1,281 treatment-switched patients, 1,077 (84.1%) had received intravitreal anti-VEGF agents other than IVT-AFL (95.6% of whom had received ranibizumab) and 322 (25.1%) had received photodynamic therapy. The most common reason for switching to IVT-AFL from other anti-VEGF agents was lack of effectiveness (882 patients, 81.9%).

**Table 3:** Incidence of important safety specifications.

Safety analysis set (n = 3,872)	Patients, n (%)			
	Adverse event		Adverse drug reaction	
	Nonserious	Serious	Nonserious	Serious
Important risks identified				
Inflammatory intraocular response	1 (0.03)	0 (0.00)	1 (0.03)	0 (0.00)
Increased intraocular pressure	20 (0.52)	2 (0.05)	15 (0.39)	1 (0.03)
Retinal tear and retinal detachment	6 (0.15)	2 (0.05)	5 (0.13)	1 (0.03)
Traumatic cataract	1 (0.03)	1 (0.03)	0 (0.00)	1 (0.03)
Important potential risk				
Arterial thromboembolic events	0 (0.00)	17 (0.44)	0 (0.00)	15 (0.39)

### Safety outcomes

#### Treatment status

In the SAF (n = 3,872), the observation period was 801.7 ± 412.5 (median: 1,035; range: 1–2,044) days. The number of injections through month 36 was 7.1 ± 5.8, the mode of the total number of injections was 3, which accounted for 22.2% of cases (n = 861). The number of injections was similar between the treatment-naïve (7.0 ± 5.5) and treatment-switched (7.5 ± 6.2) patients. The number of patients with concomitant therapy during the 36-month follow-up period was 293 (7.6%), of which 134 (3.5%) received photodynamic therapy.

#### Ocular and nonocular safety events during 36-month follow-up

Adverse events (AEs) occurred in 222 patients (5.73%) over the 36 months, with ocular AEs and nonocular AEs occurring in 159 (4.11%) and 70 (1.81%) patients, respectively. Among the AEs, ADRs were reported in 107 patients (2.76%), with ocular ADRs in 80 (2.07%) and nonocular ADRs in 28 (0.72%) patients (Table 2). The incidence of each safety event is shown in supplemental Table 4.

#### Prespecified safety specifications related to IVT-AFL treatment

One case (0.03%) of intraocular inflammatory response judged to be ADR was iritis (nonserious), which developed on day 2 of the second IVT-AFL treatment in a 58-year-old male patient (Table 3). This patient received a single treatment of bromfenac sodium hydrate and cefmenoxime hydrochloride and iritis resolved on day 12.

ADRs of either ocular hypertension (OH) or increased intraocular pressure (IOP) were reported in 7 (0.18%) and 11 (0.28%) of patients, respectively, and 2 patients (0.05%) experienced both events (Table 3, Supplemental Table 4). Among patients with recorded IOP (n = 2,917), a greater proportion of patients with the comorbidity or medical history of glaucoma and/or OH experienced an increase in IOP of at least 10 mm Hg from baseline, compared with eyes without such comorbidity or medical history (23/206 [11.17%] and 88/2,711 [3.25%], respectively). The proportion of eyes with IOP elevated to above 20 mm Hg was also greater in those with such comorbidity or medical history (45/206, 21.84%) compared to those without (260/2,711 [9.59%]).

ATEs as important ADRs were reported in 15 patients (0.39%, Table 3), cerebrovascular infarction in 9 patients (0.23%), myocardial infraction in 3 patients (0.08%), and cerebral infarction, lacunar infraction, and amaurosis fugax in 1 patient each (0.03%). All 15 patients who experienced ATEs as ADRs were aged 65 years or older. Of these, 5 patients had comorbidity (hypertension, diabetes mellitus, and/or hyperlipidaemia). Among those who developed ATEs, including both as ADRs and AEs, 16 patients (94.1%) did not have any medical history of ATEs.

Outcomes of death judged to be ADR-related were reported in 5 patients (3 cases of death, 1 case of cardiac death and 1 case of cardiac disorder; Supplemental Table 4): an 83-year-old man with a history of hypertension, an 83-year-old woman with comorbid renal dysfunction and diabetes (in these 2 cases, the number of days between last IVT-AFL treatment and death was unknown), a 63-year-old man without comorbidities (died 234th day from last IVT-AFL treatment), an 86-year-old man with comorbidities including diabetes (died due to sudden cardiac death 10th day from last IVT-AFL treatment) and a 66-year-old man with comorbidities of angina pectoris and atrial fibrillation (died due to cardiac disorder 9th day from first IVT-AFL treatment).

No patients were found to be pregnant during IVT-AFL treatment, and thus no embryotoxic or fetotoxic events were reported.

#### Duration from initial IVT-AFL treatment to onset of ADR

The timing of onset of major ADRs from the initial IVT-AFL treatment are shown in Fig. 1; not only ADRs classified as “important risks” but those occurring in 0.1% of patients or more are shown. All cases of retinal pigment epithelial (RPE) tear (7 patients [100%]) and macular hole (3 patients [100%]) and most cases of retinal haemorrhage (6/7 patients [85.7%]) and vitreous haemorrhage (3/4 patients [75.0%]) occurred within 6 months of initial treatment, whereas all cases of retinal tear (2 patients [100%]) and most cases of cataract (14/16 patients [87.5%]), OH (5/7 patients [71.4%]), and increased IOP (8/11 patients [72.7%]) developed between 6 and 36 months.

As for systemic ADRs, most cases of cerebral infarction (6/9 patients [66.7%]) occurred between 6 and 36 months after initial IVT-AFL treatment. All cases of myocardial infarction (3 patients) occurred between 3 and 6 months.

#### Reasons for treatment discontinuation

In the SAF, the treatment retention rates at 12, 24, and 36 months were 78.4%, 66.0%, and 52.9%, respectively. The main reasons for treatment discontinuation over the 36 months were achievement of treatment goal (504 patients, 27.6%), loss of visit (492 patients, 27.0%), and referral to another hospital (423 patients, 23.2%). AEs accounted for 2.5% of the reasons for treatment discontinuation (Supplemental Table 5).

### Effectiveness outcomes

The number of injections through month 36 was 7.3 ± 5.8, 7.2 ± 5.5, and 7.7 ± 6.2 for all patients in the EAS (n = 3,684), treatment-naïve patients (n = 2,393), and treatment-switched patients (n = 1,232), respectively. By the end of the study period, 1,697 patients (46.1%) in the EAS had discontinued the treatment.

**Figure 2:**
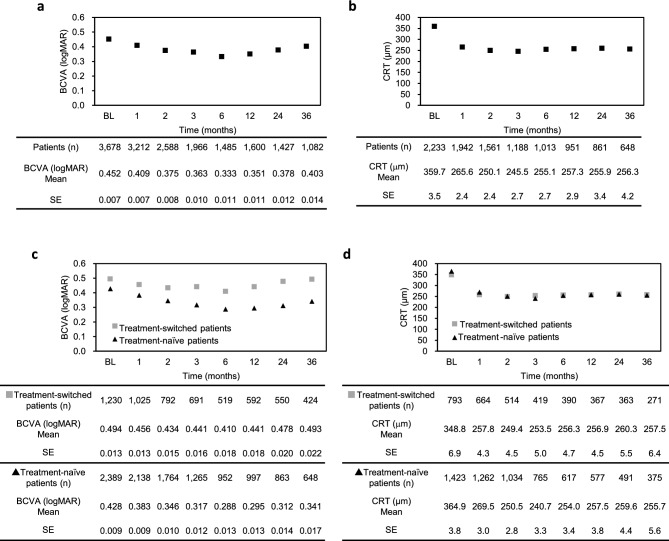
Changes in observed logMAR BCVA and CRT over the 36-month study period in all patients with nAMD (a and b, respectively) and in the treatment-naïve and treatment-switched subgroups (c and d) after starting IVT-AFL treatment. Data shown are mean values. *BCVA* best-corrected visual acuity, *BL* baseline, *CRT* central retinal thickness, *IVT-AFL* intravitreal aflibercept, *logMAR* logarithm of the minimum angle of resolution, *SE* standard error.

Mean BCVA and central retinal thickness (CRT) numerically improved over the 36 months compared with baseline (Fig. 2a,b), and similar trends were observed in the last observation carried forward (LOCF) analysis (supplemental Figure 2a and b). Although CRT was comparable between treatment-naïve and treatment-switched patients, BCVA was better in the treatment-naïve patients at every time point (Fig. 2c,d, supplemental Figure 2c and d).

## Discussion

This regulatory postmarketing surveillance study conducted in Japan was a large-scale, prospective, 36-month, observational study of 3,872 patients with nAMD who newly started IVT-AFL treatment that was designed to investigate the safety profile and effectiveness in clinical practice. The mean number of injections over the 36 months was lower than that of the 2-year results of the VIEW trials^7^. The safety findings were consistent with the known safety profile of IVT-AFL treatment in patients with nAMD in interventional studies^7,15,16^ and in long-term (24–48 months) observational studies^8,17,18^, and no new or unexpected safety signals were observed.

Patients were predominantly male (68.4%) and 40.4% had PCV, which was comparable to previous reports from Japan^2,3,19,20^. As many as 41.7% of patients had a baseline BCVA better than 0.301 logMAR. In this surveillance, 73.2% of the patients were aged 70 years or older, which is consistent with the prevalence of nAMD stratified by age group in Japan and other Asian countries^2,21^, suggesting that older adults actively received IVT-AFL treatment. As a whole, these patient baseline characteristics generally reflect those in clinical practice in Japan during the periods of enrolment of 2012–2015, as supported by recent reports^22,23^.

In this study, 1 case of nonserious intraocular inflammatory response was reported. This incidence of 0.03% is consistent with previous reports of IVT-AFL treatment^7,13,15,16,24,25^. Increased IOP after intravitreal anti-VEGF injection is a potential effect^26^. The incidence of OH and increased IOP in this cohort was 0.52% (Table 3) and is comparable to that reported previously^7^. Also, increased IOP was more likely develop during IVT-AFL treatment in eyes with comorbidities or medical history of glaucoma or OH^27^. This suggests that eyes with glaucoma or OH may be more susceptible to increased IOP following intravitreal anti-VEGF injection and that VEGF suppression might affect the trabecular meshwork function. It is reported in mice that VEGF is a paracrine regulator of the outflow facility of the trabecular meshwork^28^. Increased IOP events were observed primarily between 6 to 36 months of surveillance, suggesting that meticulous observation of IOP should occur with long-term treatment, particularly for eyes with glaucoma or OH.

Regarding systemic safety, despite the longer observation period, the incidence of ATEs was below or comparable to that reported in previous prospective trials (0.9–3.6% over 2 years)^7,15,16^ or in a population-based retrospective study among patients who received intravitreal injections of ranibizumab or aflibercept (72.75 ATEs per 1,000 person-years)^29^. Of note, among those who developed ATEs, 16 patients (94.1%) did not have any medical history of ATEs. These results suggest the need for careful observation regardless of the presence or absence of a medical history of ATEs. Moreover, this surveillance study provides important findings about the timing of ADR occurrence in relation to the start of initial treatment, although some events were not included in the prespecified important safety specification (Table 3). RPE tear, macular hole, and retinal or vitreous haemorrhage were more likely to develop within 6 months. A previous study reported that approximately 70% of RPE tears developed within 6 months of initial treatment^30^. RPE tear^30,31^ and macular hole^32^ after intravitreal anti-VEGF treatment may be secondary to choroidal neovascular membrane contraction, which can occur relatively early. In contrast, most cases of cataract, OH, increased IOP, and cerebral infarction occurred between 6 and 36 months in the present study. The case of cataracts increased extremely after 12 months; it is possible that cataract surgery was performed at a time when nAMD had improved and stabilized after 12 months of treatment, given that 27.6% of patients had cataract at baseline (Supplemental Table 2) and that cataract surgery has been reported to exacerbate nAMD^33^. These results can help to inform both physicians and patients about safety issues in the long-term management of nAMD.

Regarding effectiveness, anatomical outcomes improved in both the treatment-naïve and treatment-switched cohorts, while functional outcomes improved in the treatment-naïve cohort only, which is consistent with previous reports^34,35^. Patients with baseline BCVA better than 0.301 logMAR accounted for 41.7% of cases, and most had BCVA 0.152 logMAR or better. Thus, the ceiling effect may have impacted the observed change in BCVA. Nevertheless, BCVA improvement was observed, particularly in the treatment-naïve patients, consistent with that reported in previous studies of patients with relatively good baseline BCVA who received IVT-AFL treatment^8,9^. Although the current study was at clinical setting and the treatment protocol was not stipulated, the long-term effectiveness data was consistent with the previous data in which the treatment protocol was well-controled^36,37^.

Approximately half of all patients discontinued follow-up before 36 months, suggesting that it is challenging to maintain long-term treatment in clinical practice. However, the most common reason for treatment discontinuation was achievement of the treatment goal (27.6%), and insufficient effect of treatment and experiencing an adverse event accounted for only 7.4% and 2.5% of discontinuations, respectively. Those patients who discontinued treatment due to ‘loss of visit’ or ‘were referred to another hospital’ may have included both improved and worsened patients, while many of the patients who returned to the original clinics would have achieved a treatment goal with stabilized nAMD conditions, given that all the treating clinicians in the surveillance sites were retina specialists or those who were conducted by them. Most patients discontinued follow-up for relatively positive reasons, nevertheless, clinicians should strongly recommend that even these patients continue follow-up visits for early detection of recurrence and for avoiding undertreatment.

The strength of this study was the design, including its 36-month, multicentre, observational, prospective nature with 3,872 patients with nAMD newly starting IVT-AFL treatment. However, there are also some important limitations. First, diagnosis and treatment decisions were left to the clinicians’ discretion, which means we cannot rule out patient selection bias. Second, the treatment regimen, as well as retreatments, was decided by individual clinicians. Finally, this surveillance was conducted as a regulatory agency-mandated study, and therefore the statistical analyses were only exploratory and descriptive.

In summary, this 36-month regulatory surveillance showed acceptable tolerability and effectiveness for IVT-AFL treatment administered in clinical practice for patients with nAMD in Japan. Surveillance data showed that RPE tear and other vitreo-retinal events are more likely to develop early after starting treatment, whereas increased IOP and cerebral infarction are more likely to develop relatively later, emphasising the importance of close and continuous monitoring over the long term in nAMD patients receiving IVT-AFL treatment.

## Methods

### Study design

This 36-month, prospective, observational, postmarketing surveillance study in patients with nAMD was conducted at 177 sites in Japan from December 2012 to December 2018. The details of the 177 sites were 60 university hospitals, 67 general hospitals, and 50 clinics, all of which had retinal specialists. Patients were registered from December 2012 to June 2015.

An electronic data capture system with a central registration method was used for the surveillance component. Study physicians registered patients, confirmed the initial injections, and entered the results at each time point up to 36 months. Observation was terminated if either of the following events occurred: (1) discontinuation of IVT-AFL treatment for any reason or (2) no visit/contact on the scheduled visit date or 4 months after the last visit. The planned number of patients to be included was 4,000, to allow for detection of at least one occurrence of an ATE, based on the reported incidence of the event (0.05%) in the VIEW trials^7^ at 85% probability.

This surveillance was conducted in accordance with Good Post-Marketing Study Practice^38^ and in accordance with the principles of the Declaration of Helsinki and the International Conference on Harmonisation. No institutional review board (IRB) approval is formally required for postmarketing surveillance as the regulatory-imposed study in Japan. Therefore, the IRB for this entire study has not been set up.

### Patients and treatment

Patients diagnosed with nAMD were enrolled after the investigator decided to initiate IVT-AFL treatment. Patients who were previously treated with IVT-AFL were excluded. If a patient had bilateral nAMD, the eye that received the first dose of IVT-AFL was included in the analysis. If treatment began in both eyes on the same day, the eye with the lower BCVA at baseline was selected; if BCVA in both eyes was the same, the right eye was included. Methods for diagnosis was not stipulated in the protocol and were left to the decision of treating physician, while fluorescein and indocyanine green angiographies, and optical coherence tomography (OCT) with or without OCT angiography are mainly used in Japan.

After the first injection of 2 mg IVT-AFL, clinicians determined the need for repeated injections. Strict retreatment criteria were not stipulated in the protocol of the current study in a real-world clinical practice, while retreatment is commonly decided by presence of macular fluid in Japan. The inter-injection interval was at least 1 month, per manufacturer guidelines^5^.

### Outcome measures

The primary outcomes were the occurrence of ADRs and of AEs. The events were coded based on Medical Dictionary for Regulatory Activities version 23.0^39^. In accordance with the requirements from the Pharmaceutical and Medical Devices Agency in Japan, safety specifications (Supplemental Table 1), which consist of ‘important risks identified’ and ‘important potential risks’ defined by the Japan RMP, were also investigated.

The other prespecified outcomes investigated were number of injections received, treatment retention rate, reasons for treatment discontinuation, duration from initial IVT-AFL treatment to ADR occurrence, BCVA, ocular findings including intraocular pressure and fundus findings, and CRT measured using optical coherence tomography at each time.

Authors did not assess the low clinical records; patient data were de-identified as anonymized information before the data registration and analyses.

### Statistical analysis

The statistical analyses were exploratory and descriptive. Categorical variables were summarised as frequency or proportion and continuous variables as fundamental statistics. Data are expressed as the mean ± standard deviation. All statistical analyses were performed using SAS version 9.4 (SAS Institute Inc., Cary, NC).

Patients who received at least one IVT-AFL injection were included in the SAF. Those who had at least one available measurement of BCVA or CRT were included in the EAS. The effectiveness data were analyzed using both the observed values and the values imputed by LOCF analysis.

### Ethics statements

This surveillance was conducted as a regulatory-mandated study in compliance with the Good Post-marketing Study Practice (GPSP) and Good Vigilance Practice of the Ministry of Health, Labour, and Welfare in Japan and is registered at ClinicalTrials.gov (NCT01756248). Informed consent was not obtained in this surveillance because the Government Authority decreed that Japanese postmarketing surveillance does not require informed consent.

## Supplementary Information

Supplementary Information.

## Data Availability

Availability of the data underlying this publication will be determined according to Bayer’s commitment to the EFPIA/PhRMA “Principles for responsible clinical trial data sharing”. This pertains to scope, time point, and process of data access. As such, Bayer commits to sharing upon request from qualified scientific and medical researchers patient-level clinical trial data, study-level clinical trial data, and protocols from clinical trials in patients for medicines and indications approved in the United States (US) and European Union (EU) as necessary for conducting legitimate research. This applies to data on new medicines and indications that have been approved by the EU and US regulatory agencies on or after January 1, 2014. Interested researchers can use www.clinicalstudydatarequest.com to request access to anonymised patient-level data and supporting documents from clinical studies to conduct further research that can help advance medical science or improve patient care. Information on the Bayer criteria for listing studies and other relevant information is provided in the Study sponsors section of the portal. Data access will be granted to anonymised patient-level data, protocols, and clinical study reports after approval by an independent scientific review panel. Bayer is not involved in the decisions made by the independent review panel. Bayer will take all necessary measures to ensure that patient privacy is safeguarded. The available data shown above is also provided, when the request is sent to the corresponding author with appropriate reason.
