# Supplementary material for: Long-term surveillance provides real-world evidences of safety and effectiveness in intravitreal aflibercept treatment for age-related macular degeneration
Source: Sci Rep. 2023 Jun 30;13:10597. doi: 10.1038/s41598-023-37584-1 (PMC10313657; doi:10.1038/s41598-023-37584-1)
Supplement: Supplementary file 1 — Supplementary Information. [file 41598_2023_37584_MOESM1_ESM.pdf]

Title

Long-term surveillance provides real-world evidences of safety and effectiveness in intravitreal aflibercept treatment for age-related macular degeneration

Journal

Scientific Reports

Authors and Affiliations

Yoko Ozawa[1,2,3, 4], Kazuhiro Ohgami[4], Koji Sasaki[4], Kazufumi Hirano[4], Toshiyuki Sunaya[5]

1. Department of Clinical Regenerative Medicine Eye Center, Fujita Medical Innovation Center Tokyo, Fujita Health University School of Medicine, Tokyo, Japan
2. Department of Ophthalmology, St. Luke's International Hospital, Tokyo, Japan
3. Department of Ophthalmology, St. Luke's International University, Tokyo, Japan
4. Department of Ophthalmology, Keio University School of Medicine, Tokyo, Japan
5. Medical Affairs & Pharmacovigilance, Bayer Yakuhin, Ltd., Osaka, Japan
6. Research & Development Japan, Bayer Yakuhin, Ltd., Osaka, Japan

Corresponding Author

Yoko Ozawa, MD, PhD, Department of Clinical Regenerative Medicine Eye Center, Fujita Medical Innovation Center Tokyo, Fujita Health University School of Medicine, Tokyo, Japan

ozawa@a5.keio.jp, yoko.ozawa@fujita-hu.ac.jp

Supplemental Table 1. Safety specification definitions

**Important risks identified**	
**Intraocular inflammatory response**	Anterior chamber cell, anterior chamber fibrin, anterior chamber flare, anterior chamber inflammation, aqueous fibrin, autoimmune uveitis, candida endophthalmitis, choroiditis, chorioretinitis, cyclitis, endophthalmitis, eye infection, eye infection bacterial, eye infection chlamydial, eye infection fungal, eye infection intraocular, eye infection staphylococcal, eye inflammation, hypopyon, infectious iridocyclitis, infective iritis, infective uveitis, iridocyclitis, iritis, mycotic endophthalmitis, noninfectious endophthalmitis, noninfective chorioretinitis, pseudoendophthalmitis, uveitis, vitreal cells, vitreous fibrin, vitritis, necrotising retinitis
**Increased intraocular pressure**	Intraocular pressure increased, ocular hypertension
**Retinal tear and retinal detachment**	Macular detachment, retinal tear, retinal detachment, rhegmatogenous retinal detachment, serous retinal detachment, tractional retinal detachment
**Traumatic cataract**	Atopic cataract, cataract, cataract cortical, cataract diabetic, cataract nuclear, cataract operation, cataract subcapsular, cataract traumatic, intraocular lens implant, lens capsulotomy, lens discoloration, lens extraction, lenticular injury, lenticular opacities, lenticular operation, posterior lens capsulotomy, radiation cataract, toxic cataract
**Important potential risk**	
**Arterial thromboembolic events**	[SMQ] Ischaemic central nervous system vascular conditions (SMQ) [Narrow]<br>[SMQ] Ischaemic heart disease (SMQ) [Broad]

Note: Events were coded based on Medical Dictionary for Regulatory Activities version 23.0.
SMQ standardised MedDRA queries.

Supplemental Table 2. Comorbidities at baseline

	Patients, n (%)
**Safety analysis set**	3,872 (100)
**Ocular***	1,309 (33.8)
**Cataract**	1,069 (27.6)
**Glaucoma**	154 (4.0)
**Conjunctivitis**	99 (2.6)
**Ocular hypertension**	23 (0.6)
**Retinal pigment epitheliopathy**	11 (0.3)
**Uveitis**	5 (0.1)
**Endophthalmitis**	1 (0.0)
**Other**	197 (5.1)
**Nonocular***	1,391 (35.9)
**Hypertension**	940 (24.3)
**Diabetes mellitus**	331 (8.5)
**Hyperlipidaemia**	237 (6.1)
**Angina pectoris**	48 (1.2)
**Renal impairment**	41 (1.1)
**Impaired liver function**	31 (0.8)
**Old cerebral infarction**	20 (0.5)
**Old myocardial infarction**	5 (0.1)
**Intracerebral haemorrhage**	2 (0.1)
**Subarachnoid haemorrhage**	1 (0.0)
**Acute myocardial infarction**	1 (0.0)
**Other**	411 (10.6)

*Including overlap.

Supplemental Table 3. Medical history

	Patients, n (%)
**Safety analysis set**	3,872 (100)
**Ocular***	1,222 (31.6)
**Cataract**	1,036 (26.8)
**Glaucoma**	57 (1.5)
**Conjunctivitis**	40 (1.0)
**Retinal pigment epitheliopathy**	22 (0.6)
**Uveitis**	12 (0.3)
**Ocular hypertension**	10 (0.3)
**Endophthalmitis**	0 (0.0)
**Other**	172 (4.4)
**Nonocular***	890 (23.0)
**Hypertension**	326 (8.4)
**Diabetes mellitus**	122 (3.2)
**Old cerebral infarction**	70 (1.8)
**Hyperlipidaemia**	45 (1.2)
**Angina pectoris**	45 (1.2)
**Old myocardial infarction**	26 (0.7)
**Renal impairment**	20 (0.5)
**Impaired liver function**	19 (0.5)
**Acute myocardial infarction**	14 (0.4)
**Cerebral haemorrhage**	8 (0.2)
**Subarachnoid haemorrhage**	5 (0.1)
**Other**	432 (11.2)

*Including overlap.

Supplemental Table 4. Incidence of safety events

Safety analysis set (n = 3,872)	Patients, n (%)*			
	AE	SAE	ADR	SADR
**Any ocular event**	159 (4.11)	45 (1.16)	80 (2.07)	23 (0.59)
**Eye disorders**	144 (3.72)	45 (1.16)	71 (1.83)	23 (0.59)
**Cataract**	29 (0.75)	8 (0.21)	16 (0.41)	3 (0.08)
**Retinal haemorrhage**	20 (0.52)	10 (0.26)	7 (0.18)	5 (0.13)
**Vitreous haemorrhage**	13 (0.34)	9 (0.23)	4 (0.10)	3 (0.08)
**Ocular hypertension**	11 (0.28)	2 (0.05)	7 (0.18)	1 (0.03)
**Retinal pigment epithelial tear**	7 (0.18)	6 (0.15)	7 (0.18)	6 (0.15)
**Dry eye**	6 (0.15)	0	2 (0.05)	0
**Glaucoma**	6 (0.15)	0	2 (0.05)	0
**Posterior capsule opacification**	5 (0.13)	0	1 (0.03)	0
**Macular hole**	4 (0.10)	3 (0.08)	3 (0.08)	2 (0.05)
**Conjunctival haemorrhage**	4 (0.10)	0	3 (0.08)	0
**Visual acuity reduced**	4 (0.10)	0	1 (0.03)	0
**Retinal detachment**	3 (0.08)	1 (0.03)	3 (0.08)	1 (0.03)
**Eye pain**	3 (0.08)	0	3 (0.08)	0
**Retinal tear**	3 (0.08)	0	2 (0.05)	0
**Eye pruritus**	3 (0.08)	0	1 (0.03)	0
**Conjunctivitis allergic**	3 (0.08)	0	0	0
**Choroidal haemorrhage**	2 (0.05)	2 (0.05)	1 (0.03)	1 (0.03)
**Macular fibrosis**	2 (0.05)	0	1 (0.03)	0
**Amaurosis fugax**	1 (0.03)	1 (0.03)	1 (0.03)	1 (0.03)
**Retinal vein occlusion**	1 (0.03)	1 (0.03)	1 (0.03)	1 (0.03)
**Corneal degeneration**	1 (0.03)	1 (0.03)	0	0
**Lens dislocation**	1 (0.03)	1 (0.03)	0	0
**Rhegmatogenous retinal detachment**	1 (0.03)	1 (0.03)	0	0
**Subretinal haematoma**	1 (0.03)	1 (0.03)	0	0
**Vitreous opacities**	1 (0.03)	1 (0.03)	0	0
**Abnormal sensation in eye**	1 (0.03)	0	1 (0.03)	0
**Chorioretinal atrophy**	1 (0.03)	0	1 (0.03)	0
**Corneal disorder**	1 (0.03)	0	1 (0.03)	0
**Corneal erosion**	1 (0.03)	0	1 (0.03)	0
**Iritis**	1 (0.03)	0	1 (0.03)	0
**Macular oedema**	1 (0.03)	0	1 (0.03)	0

**Photophobia**	1 (0.03)	0	1 (0.03)	0
**Serous retinal detachment**	1 (0.03)	0	1 (0.03)	0
**Vision blurred**	1 (0.03)	0	1 (0.03)	0
**Age-related macular degeneration**	1 (0.03)	0	0	0
**Aniseikonia**	1 (0.03)	0	0	0
**Asthenopia**	1 (0.03)	0	0	0
**Blepharitis**	1 (0.03)	0	0	0
**Endocrine ophthalmopathy**	1 (0.03)	0	0	0
**Hypotony of eye**	1 (0.03)	0	0	0
**Neovascular age-related macular degeneration**	1 (0.03)	0	0	0
**Normal tension glaucoma**	1 (0.03)	0	0	0
**Photopsia**	1 (0.03)	0	0	0
**Punctate keratitis**	1 (0.03)	0	0	0
**Retinal thickening**	1 (0.03)	0	0	0
**Subretinal fluid**	1 (0.03)	0	0	0
**Vitreous detachment**	1 (0.03)	0	0	0
**Investigations**	13 (0.34)	0	11 (0.28)	0
**Intraocular pressure increased**	13 (0.34)	0	11 (0.28)	0
**Infections and infestations**	5 (0.13)	0	0	0
**Conjunctivitis**	4 (0.10)	0	0	0
**Adenoviral conjunctivitis**	1 (0.03)	0	0	0
**Surgical and medical procedures**	1 (0.03)	0	0	0
**Cataract operation**	1 (0.03)	0	0	0
**Any systemic event**	70 (1.81)	50 (1.29)	28 (0.72)	21 (0.54)
**Nervous system disorders**	19 (0.49)	16 (0.41)	14 (0.36)	12 (0.31)
**Cerebral infarction**	9 (0.23)	9 (0.23)	9 (0.23)	9 (0.23)
**Cerebellar infarction**	1 (0.03)	1 (0.03)	1 (0.03)	1 (0.03)
**Lacunar infarction**	1 (0.03)	1 (0.03)	1 (0.03)	1 (0.03)
**Peripheral nerve palsy**	1 (0.03)	1 (0.03)	1 (0.03)	1 (0.03)
**Cerebral haemorrhage**[†]	1 (0.03)	1 (0.03)	0	0
**Cervical cord compression**	1 (0.03)	1 (0.03)	0	0
**Subarachnoid haemorrhage**[†]	1 (0.03)	1 (0.03)	0	0
**Syncope**	1 (0.03)	1 (0.03)	0	0
**Dizziness**	1 (0.03)	0	1 (0.03)	0
**Hypoaesthesia**	1 (0.03)	0	1 (0.03)	0
**Presyncope**	1 (0.03)	0	0	0

**Neoplasms benign, malignant, and unspecified (including cysts and polyps)**	12 (0.31)	12 (0.31)	0	0
**Angiosarcoma**	1 (0.03)	1 (0.03)	0	0
**Breast cancer**	1 (0.03)	1 (0.03)	0	0
**Gastric cancer**	1 (0.03)	1 (0.03)	0	0
**Gastric cancer stage IV**	1 (0.03)	1 (0.03)	0	0
**Lung neoplasm malignant**	1 (0.03)	1 (0.03)	0	0
**Lymphoma**	1 (0.03)	1 (0.03)	0	0
**Metastases to central nervous system**	1 (0.03)	1 (0.03)	0	0
**Metastases to liver**	1 (0.03)	1 (0.03)	0	0
**Metastases to lung**	1 (0.03)	1 (0.03)	0	0
**Metastases to peritoneum**[†§]	1 (0.03)	1 (0.03)	0	0
**Pancreatic carcinoma**[†]	1 (0.03)	1 (0.03)	0	0
**Plasma cell myeloma**[†]	1 (0.03)	1 (0.03)	0	0
**Renal cancer**	1 (0.03)	1 (0.03)	0	0
**Small cell carcinoma**	1 (0.03)	1 (0.03)	0	0
**General disorders and administration site conditions**	9 (0.23)	6 (0.15)	4 (0.10)	4 (0.10)
**Death**[†‡]	5 (0.13)	5 (0.13)	3 (0.08)	3 (0.08)
**Pyrexia**	2 (0.05)	0	0	0
**Cardiac death**[†‡]	1 (0.03)	1 (0.03)	1 (0.03)	1 (0.03)
**Gait disturbance**	1 (0.03)	0	0	0
**Cardiac disorders**	8 (0.21)	7 (0.18)	5 (0.13)	4 (0.10)
**Myocardial infarction**	4 (0.10)	4 (0.10)	3 (0.08)	3 (0.08)
**Cardiac disorder**[†‡]	1 (0.03)	1 (0.03)	1 (0.03)	1 (0.03)
**Angina pectoris**	1 (0.03)	1 (0.03)	0	0
**Sinus node dysfunction**	1 (0.03)	1 (0.03)	0	0
**Bradycardia**	1 (0.03)	0	1 (0.03)	0
**Hepatobiliary disorders**	3 (0.08)	1 (0.03)	1 (0.03)	0
**Cholecystitis**	1 (0.03)	1 (0.03)	0	0
**Hepatic function abnormal**	1 (0.03)	0	1 (0.03)	0
**Cholangitis**	1 (0.03)	0	0	0
**Infections and infestations**	3 (0.08)	2 (0.05)	0	0
**Pneumonia**	1 (0.03)	1 (0.03)	0	0
**Pulmonary tuberculosis**	1 (0.03)	1 (0.03)	0	0
**Herpes zoster disseminated**	1 (0.03)	0	0	0
**Investigations**	3 (0.08)	0	2 (0.05)	0

**Blood pressure increased**	2 (0.05)	0	2 (0.05)	0
**Tumor marker increased**	1 (0.03)	0	0	0
**Musculoskeletal and connective tissue disorders**	3 (0.08)	1 (0.03)	1 (0.03)	1 (0.03)
**Joint range of motion decreased**	1 (0.03)	1 (0.03)	1 (0.03)	1 (0.03)
**Muscular weakness**	1 (0.03)	0	0	0
**Pain in extremity**	1 (0.03)	0	0	0
**Ear and labyrinth disorders**	2 (0.05)	0	1 (0.03)	0
**Hypoacusis**	1 (0.03)	0	1 (0.03)	0
**Tinnitus**	1 (0.03)	0	0	0
**Gastrointestinal disorders**	2 (0.05)	1 (0.03)	1 (0.03)	0
**Ileus**[†§]	1 (0.03)	1 (0.03)	0	0
**Colitis ischaemic**	1 (0.03)	0	1 (0.03)	0
**Injury, poisoning, and procedural complications**	2 (0.05)	1 (0.03)	0	0
**Stab wound**[†]	1 (0.03)	1 (0.03)	0	0
**Upper limb fracture**	1 (0.03)	0	0	0
**Renal and urinary disorders**	2 (0.05)	2 (0.05)	0	0
**Calculus bladder**	1 (0.03)	1 (0.03)	0	0
**Chronic kidney disease**[†]	1 (0.03)	1 (0.03)	0	0
**Surgical and medical procedures**	2 (0.05)	1 (0.03)	1 (0.03)	1 (0.03)
**Hospitalisation**	1 (0.03)	1 (0.03)	1 (0.03)	1 (0.03)
**Vitrectomy**	1 (0.03)	0	0	0
**Vascular disorders**	2 (0.05)	1 (0.03)	0	0
**Aortic aneurysm**	1 (0.03)	1 (0.03)	0	0
**Hypertension**	1 (0.03)	0	0	0
**Respiratory, thoracic, and mediastinal disorders**	1 (0.03)	1 (0.03)	0	0
**Pneumothorax**	1 (0.03)	1 (0.03)	0	0
**Skin and subcutaneous tissue disorders**	1 (0.03)	0	1 (0.03)	0
**Rash**	1 (0.03)	0	1 (0.03)	0
**Congenital, familial, and hereditary disorders**	1 (0.03)	0	0	0
**Color deficiency**	1 (0.03)	0	0	0
**Immune system disorders**	1 (0.03)	0	0	0
**Seasonal allergy**	1 (0.03)	0	0	0

*Including patients with multiple events.

†Outcomes of death was reported in 14 patients in total, in which 5 cases were judged as ADR (‡)

§These two events were reported in a single patient.

Supplemental Table 5. Reasons for treatment discontinuation

Safety analysis set (n=3,872)	Treatment period*			
	Total	1-12 months	13-24 months	25-36 months
**Discontinuation of treatment, n**	1,823	837	480	506
**Reason, n (%)†**				
**Achievement of treatment goal**	504 (27.6)	268 (32.0)	126 (26.3)	110 (21.7)
**Loss of visit**	492 (27.0)	185 (22.1)	112 (23.3)	126 (24.9)
**Referral to another hospital**	423 (23.2)	172 (20.5)	136 (28.3)	184 (36.4)
**Patient's wishes**	191 (10.5)	109 (13.0)	34 (7.1)	48 (9.5)
**Insufficient effect**	135 (7.4)	82 (9.8)	37 (7.7)	16 (3.2)
**Difficulty attending visit**	119 (6.5)	56 (6.7)	31 (6.5)	32 (6.3)
**Adverse event**	45 (2.5)	31 (3.7)	10 (2.1)	4 (0.8)
**Other**	81 (4.4)	41 (4.9)	21 (4.4)	19 (3.8)

*Total: +1 to +1,109 days from first date of IVT-AFL; 1-12 months: +1 to +389 days; 13-24 months: +390 to +749 days; 25-36 months: +750 to +1,109 days.

†Including patients with multiple reasons.

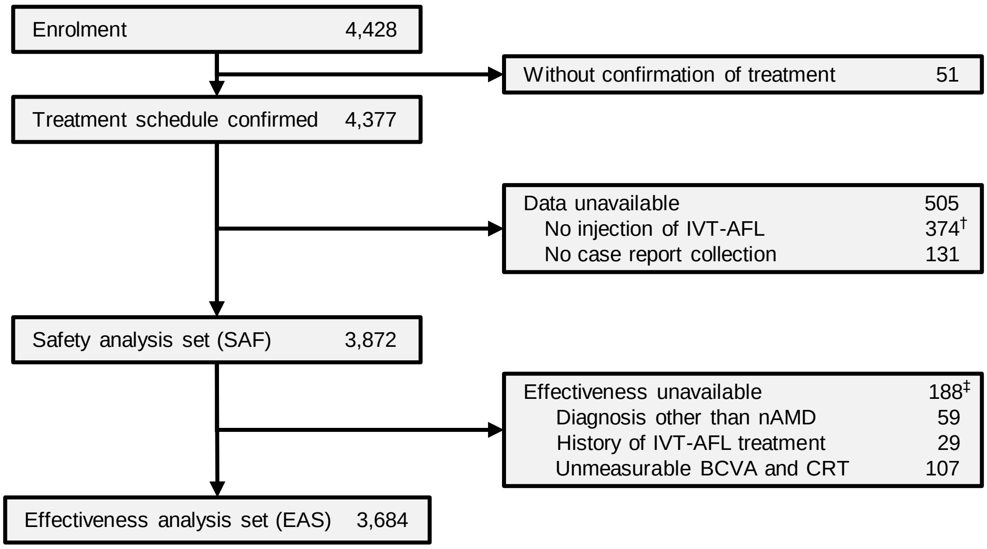

Supplemental Figure 1. Patient flow diagram.

Among the 4,428 enrolled patients, 4,377 patients were scheduled to undergo IVT-AFL by the physicians. However, 374 patients had no record for IVT-AFL, and 131 patients had no case reports, thus they were excluded from the safety analysis set (SAF). From the 3,872 patients included in the SAF, 59 patients whose diagnosis was not nAMD and 29 patients who had histories of previous IVT-AFL treatments both revealed by the case report review, and 107 patients who had no data for BCVA and CRT were excluded from the effectiveness analysis set (EAS), thus effectiveness was analyzed in 3,684 patients.

†Includes 4 patients who were found not to have started IVT-AFL after CRF collection.

‡Includes overlap.

BCVA best-corrected visual acuity, *CRF* case report form, *CRT* central retinal thickness, *IVT-AFL* intravitreal aflibercept, *nAMD,* neovascular age-related macular degeneration.

a

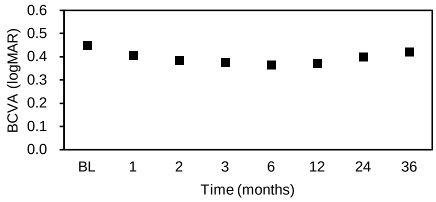

Patients (n)	3,678	3,212	3,381	3,506	3,578	3,646	3,670	3,674
BCVA (logMAR) Mean	0.452	0.409	0.387	0.379	0.368	0.373	0.403	0.423
SE	0.007	0.007	0.007	0.007	0.007	0.007	0.008	0.008

b

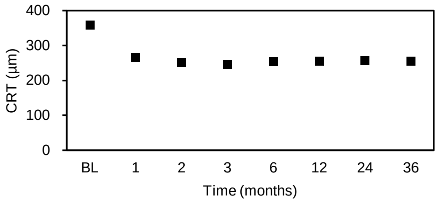

Patients (n)	2,233	1,942	2,083	2,188	2,272	2,342	2,367	2,427
CRT (µm) Mean	359.7	265.6	251.0	245.7	254.8	255.6	256.7	255.2
SE	3.5	2.4	2.1	1.93	2.2	2.2	2.2	2.2

c

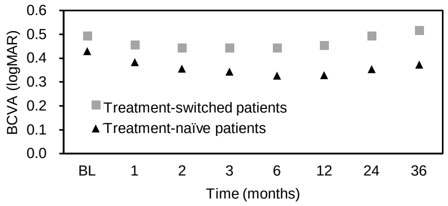

■Treatment-switched patients (n)	1,230	1,025	1,104	1,163	1,185	1,219	1,226	1,227
BCVA (logMAR) Mean	0.494	0.456	0.444	0.444	0.443	0.454	0.493	0.517
SE	0.013	0.013	0.013	0.013	0.013	0.013	0.013	0.014
▲Treatment-naïve patients (n)	2,389	2,138	2,225	2,290	2,340	2,374	2,386	2,388
BCVA (logMAR) Mean	0.428	0.383	0.355	0.342	0.326	0.328	0.354	0.371
SE	0.009	0.009	0.009	0.009	0.009	0.009	0.009	0.010

d

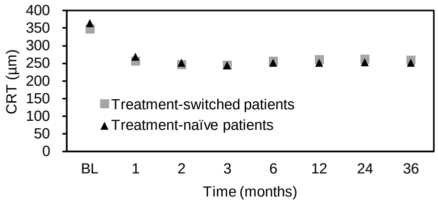

■Treatment-switched patients (n)	793	664	733	777	810	837	841	858
CRT (µm) Mean	348.8	257.8	248.4	246.7	258.8	262.1	263.4	260.8
SE	6.87	4.26	3.69	3.45	4.19	4.32	4.13	4.06
▲Treatment-naïve patients (n)	1,423	1,262	1,334	1,394	1,445	1,487	1,508	1,551
CRT (µm) Mean	364.9	269.5	252.3	244.9	252.2	251.9	253.3	252.3
SE	3.8	3.0	2.6	2.3	2.5	2.5	2.5	2.5

Supplemental Figure 2. Changes in logMAR BCVA and CRT over the 36-month study period imputed by the LOCF method in all nAMD patients (a and b, respectively) and in the treatment-naïve and treatment-switched subgroups (c and d) after starting IVT-AFL treatment.

Data shown are mean values.

BCVA best-corrected visual acuity, *BL* baseline, *CRT* central retinal thickness, *IVT-AFL* intravitreal aflibercept, *LOCF* last observation carried forward, *logMAR* logarithm of the minimum angle of resolution, *SE* standard error.
